# A scale for measuring home-based cardiac rehabilitation exercise adherence: a development and validation study

**DOI:** 10.1186/s12912-023-01426-2

**Published:** 2023-08-07

**Authors:** Zhen Yang, Yuanhui Sun, Huan Wang, Chunqi Zhang, Aiping Wang

**Affiliations:** 1 The First Affiliated Hospital of China Medical University, No.155, Nanjing North Street, Heping District, Shenyang, Liaoning Province China; 2 The First Affiliated Hospital of Jinzhou Medical University, No.2, Section 5, Renmin Street, Guta District, Jinzhou City, Liaoning Province China

**Keywords:** Home-based cardiac rehabilitation, Exercise adherence scale, Chronic heart failure, Psychometric properties, Delphi survey

## Abstract

**Background:**

The benefits of home-based cardiac rehabilitation exercise are well-established and depend on long-term adherence. However, there is no uniform and recognized cardiac rehabilitation criterion to assess home-based cardiac rehabilitation exercise adherence for patients with cardiovascular disease. This study aimed to develop a home-based cardiac rehabilitation exercise adherence scale and to validate its psychometric properties among patients with chronic heart failure.

**Methods:**

The dimensions and items of the scale were created based on grounded theory research, literature content analysis, and defined by a Delphi survey. Item analysis was completed to assess the discrimination and homogeneity of the scale. Factor analysis was adopted to explore and validate the underlying factor structure of the scale. Content validity and calibration validity were evaluated using the Delphi survey and correlation analysis, respectively. Reliability was evaluated by Cronbach’s α coefficients, split-half reliability coefficients, and test-retest reliability coefficients.

**Results:**

A scale covering four dimensions and 20 items was developed for evaluating home-based cardiac rehabilitation exercise adherence. The content validity index of the scale was 0.986. In exploratory factor analysis, a four-factor structure model was confirmed, explaining 75.1% of the total variation. In confirmatory factor analysis, the four-factor structure was supported by the appropriate fitting indexes. Calibration validity of the scale was 0.726. In terms of reliability, the Cronbach’s α coefficient of the scale was 0.894, and the Cronbach’s α coefficients of dimensions ranged from 0.848 to 0.914. The split-half reliability coefficient of the scale was 0.695. The test-retest reliability coefficient of the scale was 0.745.

**Conclusion:**

In this study, a home-based cardiac rehabilitation exercise adherence scale was developed and its appropriate psychometric properties were confirmed.

**Supplementary Information:**

The online version contains supplementary material available at 10.1186/s12912-023-01426-2.

## Introduction

Despite progress in prevention and control, the number of patients with cardiovascular disease is still on the rise due to global aging [1] Cardiovascular disease remains a leading contributor to human mortality and loss of healthy years, thus increasing the global disease burden [2]. Therefore, it is essential to explore effective interventions to improve clinical prognosis and the quality of life of patients with cardiovascular disease.

Cardiac rehabilitation aims at ensuring that patients with cardiovascular disease achieve optimal physical, mental, and social functioning through their efforts [3]. Various academic organizations recommend cardiac rehabilitation as level IA evidence for enhancing cardiopulmonary function [4, 5]. Exercise-centered cardiac rehabilitation has been shown to significantly improve clinical outcomes and reduce cardiovascular risk in patients with cardiovascular disease [6–8]. However, due to the chronic nature of the disease, long-term center-based cardiac rehabilitation exercise brings heavy time and economic costs. Home-based cardiac rehabilitation exercise has emerged as a meaningful alternative mode [9], offering similar benefits to center-based cardiac rehabilitation exercise in improving exercise endurance and clinical prognosis, promoting mental health, improving cardiopulmonary function, and reducing cardiovascular risk [10–13]. Importantly, exercise-based telehealth home cardiac rehabilitation is more cost-effective than center-based cardiac rehabilitation [14]. These benefits heavily rely on the long-term adherence of patients with cardiovascular disease to home-based cardiac rehabilitation exercise. Therefore, there is a need for a comprehensive and scientific evaluation of home-based cardiac rehabilitation exercise adherence, which is crucial for improving clinical outcomes and patients’ quality of life, and essential for clinical practice.

Currently, there is a lack of uniform and recognized criterion for assessing home-based cardiac rehabilitation exercise adherence among patients with cardiovascular disease. In existing studies, a series of relevant scales were developed and validated to assess cardiac rehabilitation preference and barriers, covering the cardiac rehabilitation inventory [15], the cardiac rehabilitation barriers scale [16], the cardiac rehabilitation preference form [17], and the information needs in cardiac rehabilitation scale [18]. However, none of these scales are suitable for assessing adherence to home-based cardiac rehabilitation exercises. In addition, self-reported exercise diaries and/or smart wearable devices were adopted to obtain exercise-related data to calculate on the basis of one aspect of the ratio (such as time, frequency) to represent the home-based cardiac rehabilitation exercise adherence, most commonly as the percentage of exercise duration to the total recommended duration [19–22]. However, such evaluation indicators are insufficient and fail to fully reflect home-based cardiac rehabilitation exercise adherence. Furthermore, the measurement indexes and their calculation formulas used in existing research are often inconsistent, which limits the credibility and comparability of research results and hinders the promotion and application of research findings. Therefore, there is an urgent need to develop a reliable tool for assessing home-based cardiac rehabilitation exercise adherence among patients with cardiovascular disease.

In a previous study [23], we explored a conceptual model of home-based cardiac rehabilitation exercise adherence using constructivist grounded theory, which revealed that seeking supports, exercise monitoring, and information feedback were essential components of home-based cardiac rehabilitation exercise adherence, in addition to rehabilitation exercise. Home-based cardiac rehabilitation exercise adherence is defined as the consistent and active engagement of patients in rehabilitation exercises within their home environment, incorporating essential elements such as seeking supports, exercise monitoring, and information feedback. This comprehensive understanding of adherence not only focuses on the performance of prescribed exercises but also recognizes the importance of supportive resources, progress tracking, and feedback mechanisms in maintaining long-term commitment and success in home-based cardiac rehabilitation programs. In this model [23], seeking supports is the initial adherence behavior, and rehabilitation exercise is the core adherence behavior, and exercise monitoring is the key adherence behavior, and information feedback is the driving adherence behavior. Therefore, this conceptual model provides a scientific and appropriate dimensional basis for the development of scales in this study.

Therefore, building on the findings of previous constructivist grounded theory research [23], this study aimed to develop a home-based cardiac rehabilitation exercise adherence scale and evaluate its psychometric properties among patients with chronic heart failure. The purpose is to identify areas of weakness in adherence and cardiac rehabilitationeate targeted interventions.

## Methods

### Participants

Eligible patients with chronic heart failure were recruited using the convenient sampling with the help of community health service workers in 4 communities in Liaoning province in mainland China. In this study, participants had to meet several inclusion cardiac rehabilitationiteria, including being 18 years of age or older, engaging in home-based cardiac rehabilitation, and voluntarily participation. In this study, home-based cardiac rehabilitation refers to the phase during which patients with chronic heart failure continue their cardiac rehabilitation at home after completing the initial rehabilitation process in a healthcare institution. The inclusion criteria was confirmed by community electronic records, community health service workers, and researchers. On the other hand, patients with various mental illnesses or had other significant organic diseases were not permitted to join. The sample size was established based on the general rule of factor analysis [24], which recommends a minimum of five respondents for each item. Ultimately, a total of 366 patients with chronic heart failure were finally enrolled in the study. The first phase of the study (item generation and revision) did not involve participants. This sample (n = 366) was used in the second phase (item evaluation and exploration) and the third phase (psychometric evaluation of the scale). In the third phase of factor analysis, samples were randomly assigned to two groups, one for exploratory factor analysis (n = 140) and the other for confirmatory factor analysis (n = 226).

### Design

**Fig. 1:**
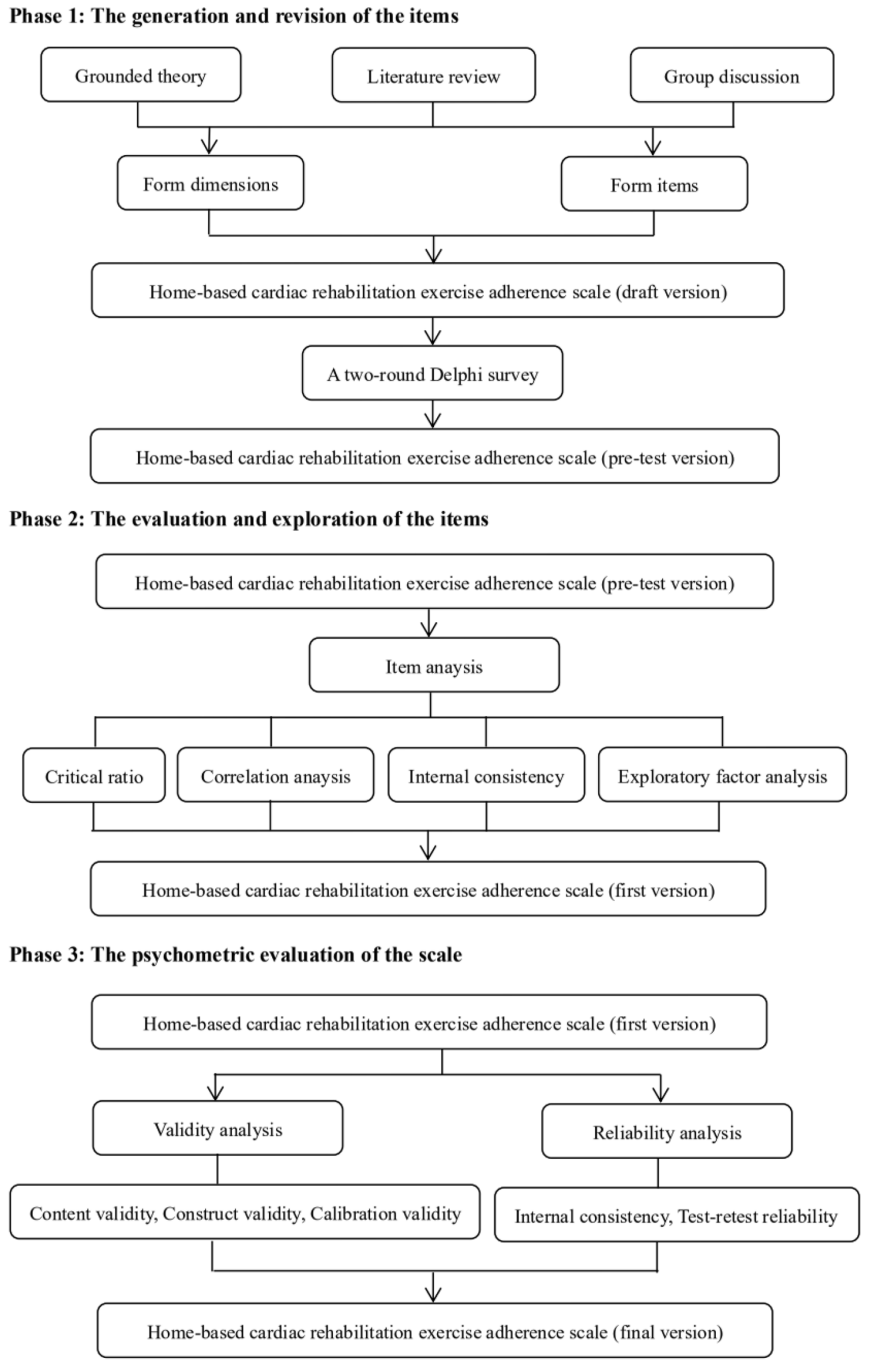
The development procedure of the home-based cardiac rehabilitation exercise adherence scale

From February to April 2023, we conducted a multi-phase study using both quantitative and qualitative approaches to develop and validate the home-based cardiac rehabilitation exercise adherence scale, which involved three phases: (a) item generation and revision; (b) item evaluation and exploration; (c) psychometric evaluation of the scale. The development process of the scale is depicted in Fig. 1.

### The generation and revision of the items

The literature review was conducted to identify relevant studies on cardiac rehabilitation exercise adherence. The review involved a content analysis approach to select meaningful sentences which were used to form an initial item pool. Additionally, based on the previous constructivist grounded theory [23], the dimensions were preliminarily determined and the item pool was supplemented. The items were then revised through a two-round Delphi survey involving eleven experts from six cities in mainland China. The selection criteria of the consultants were: (a) engaged in cardiac rehabilitation nursing for more than 15 years; (b) has intermediate professional titles or above; (c) has bachelor degree or above; (d) voluntary participation in the study. The questionnaire recovery rate was used to gauge the enthusiasm of the experts. The authority coefficient was the average of the familiarity with the field and the index judgment criteria. The degree of consensus among the experts’ opinions was determined by Kendall’s concordance coefficient, which reflected their judgment and familiarity coefficients. We set the threshold for expert enthusiasm, authority coefficient, and Kendall’s concordance coefficient at 0.70, and Kendall’s tests significance level at P < 0.05 [25]. Based on the Delphi survey results, the research team converted the item pool into a pre-test version of the scale, and the scoring system to collect participants’ responses was determined by the invited experts.

### The evaluation and exploration of the items

The items of the pre-test scale were evaluated using a range of analytical methods, including critical ratio analysis, correlation coefficient analysis, and internal consistency analysis. The samples were divided into a high group (top 27% scores) and a low group (bottom 27% scores), and the two groups of samples were analyzed to appraise the discrimination of the items. An acceptable critical ratio for each item was 3.0 or above and significant (P < 0.05) [26]. Item-total correlation coefficients were calculated to assess the applicability of the items, requiring a minimum correlation coefficient of 0.4. The homogeneity of the items was assessed using Cronbach’s α coefficient after deleting each item. Ideally, deleting any item should not increase the total Cronbach’s α coefficient. Preliminary exploratory factor analysis was adopted to explore the factor loadings to evaluate the stability of the items. The recommended minimum factor loadings were 0.4, and no cross-loadings were permitted. If any item failed to meet any of these conditions, it was excluded from the pre-test scale [27, 28].

### The psychometric evaluation of the scale

#### Content validity

The content validity of the scale was assessed by inviting seven experts. The inclusion criteria for experts were consistent with the inclusion criteria for the previous Delphi experts. A four-point Likert scoring system, which spans from one point (irrelevant) to four points (very relevant), was utilized to collect expert responses. The content validity index of the item (I-CVI) was calculated as the ratio of the number of experts ranking an item 3 or 4 points to the total number of experts. For the content validity index of the scale (S-CVI), the average of all item I-CVI scores was calculated. To meet the criteria for content validity, an I-CVI score of 0.78 or above and an S-CVI score of 0.90 or higher were required [29].

#### Construct validity

To assess the construct validity of the scale, an exploratory factor analysis (EFA) was conducted to reveal the underlying factor structure and establish consistency with the conceptual framework. Bartlett’s test of sphericity and the Kaiser-Meyer-Olkin (KMO) value were used to determine the suitability of data for EFA, with a significant result (P < 0.05) and a KMO above 0.6 as prerequisites. All common factors should account for more than 40% of the total variance [30–32]. Additionally, confirmatory factor analysis (CFA) was used to verify the factor structure obtained through EFA and grounded theory [23]. The recommended goodness of fit indices were reported in Table 1 [33, 34]. Simultaneously, the convergent and discriminant validity of the scale were assessed for construct validity. The average variance extracted (AVE) value and composite reliability (CR) value were calculated to evaluate the convergent validity. When the AVE value is above 0.50, but values above 0.40 also be acceptable, and the CR value is above 0.70, it indicates that the scale possesses appropriate convergent validity [35]. Discriminant validity was evaluated by calculating the square root of the AVE value and the correlation coefficients between factors. It is required that the square root of the AVE value be greater than the correlation coefficients between the corresponding factors [35].

**Table 1:** The reference, initial and modified fitting index values of the four-factor model

Fitting indexs	Reference value	Initial value	Modified value
Chi-square degree of freedom (χ^2^/df)	≤ 3.00	3.215	1.420
Root mean square error of approximation (RMSEA)	≤ 0.05	0.090	0.043
Goodness-of-fit index (GFI)	≥ 0.90	0.821	0.912
Adjusted goodness-of-fit index (AGFI)	≥ 0.90	0.771	0.885
Tucker lewis index (TLI)	≥ 0.90	0.880	0.977
Comparative fit index (CFI)	≥ 0.90	0.896	0.981
Incremental fit index (IFI)	≥ 0.90	0.897	0.981
Parsimonious goodness-of-fit index (PGFI)	≥ 0.70	0.641	0.703
Parsimonious normed-of-fit index (PNFI)	≥ 0.70	0.740	0.799

#### Calibration validity

To examine the calibration validity, the exercise self-efficacy scale [36] was adopted as a calibration tool for the home-based cardiac rehabilitation exercise adherence scale. The correlation coefficient between the scores on both scales was required to be 0.70 or higher to establish good calibration validity [37].

As a calibration tool, the Chinese version of the multidimensional exercise self-efficacy scale was adopted to assess patients’s confidence in adherence to exercise in this study [38]. The scale includes nine items across three dimensions, with each item being rated on a scale of 0 to 10, with 0 representing no confidence and 10 representing complete confidence. Higher scores indicate greater confidence in adhering to exercise. The Chinese version of the exercise self-efficacy scale has good psychometric properties and is widely adopted.

#### Internal consistency reliability and test-retest reliability

To evaluate the internal consistency reliability of the scale, the Cronbach’s α coefficient and the split-half reliability coefficient were calculated. Additionally, 36 participants previously surveyed were invited to complete the questionnaire again two weeks later to assess the scale’s stability across time. To establish good internal consistency, reliability, and test-retest reliability, the Cronbach’s α coefficient, the split-half reliability coefficient, and the test-retest reliability coefficient should be 0.70 or higher [39, 40].

### Data collection

In the first phase, the experts were provided with a compressed package containing informed consent and an expert consultation questionnaire via email, following a brief introduction to the study’s purpose and significance. The experts were asked to provide their feedback and suggestions within two weeks of receiving the questionnaire. In the second phase, community outpatient follow-ups were conducted for 380 chronic heart failure patients. Before participation, the patients were informed about the purpose, importance, and voluntary and anonymous nature of the study. Out of the 380 patients who were invited to participate, 366 provided anonymous responses to the questionnaires after providing consent.

### Data analysis

The data was analyzed using SPSS 26.0 and AMOS 18.0 software. The Delphi survey was conducted to revise the items and assess the content validity of the scale. In the EFA, the maximum variance rotation was adopted to explore the underlying factor structure. The structural equation model, using the maximum likelihood method, was utilized to verify the factor structure’s consistency with the theoretical expectation. To determine the scale’s reliability, internal consistency analysis and test-retest reliability analysis were performed, assessing the scale’s homogeneity and stability, respectively.

### Ethical consideration

In this study, all procedures were conducted in accordance to the Declaration of Helsinki of 1964 and its further modifications. All participants signed informed consent forms. The participants were allowed to withdraw from the study at any point and were not obligated to respond to any questions. The protocol of this study was approved by the Ethics Review Committee of the First Affiliated Hospital of China Medical University on January 27, 2023 (No. 2023. 66).

## Results

### Sociodemographic characteristics of the participants

The study included 366 chronic heart failure patients, comprising 200 males and 166 females. The average age of the participants was 66.46 ± 6.49. Nearly half of the participants had primary education (45.6%), and the majority were married (56.3%). Regarding monthly income, 49.7% of participants earned less than 3000 RMB, and 48.6% of participants had a condition duration of less than four years. The majority of participants were from urban areas (80.1%) (Table 2).

**Table 2:** Sociodemographic characteristics of the participants (n = 366)

Factors	Group	n	%
Age	＜60	48	13.1
	60 ~70	186	50.8
	＞70	132	36.1
Gender	Male	200	54.6
	Female	166	45.4
Marital status	Unmarried	24	6.6
	Married	206	56.3
	Divorced/Widowed	136	37.2
Education level	Primary education	167	45.6
	Secondary education	114	31.2
	Advanced education	85	23.2
Duration(year)	＜4	178	48.6
	4～8	144	39.3
	＞8	44	12.0
Monthly income(rmb)	＜3000	182	49.7
	3000 ~6000	126	34.4
	＞6000	58	15.8
Location	Village	73	19.9
	City	293	80.1

### The generation and revision of the items

In the literature review, a pool of 46 items was generated following content analysis. Based on the previous grounded theory, the item pool was further supplemented and four dimensions were preliminarily confirmed. On this basis, a 45-item pool was generated as a means of further selecting items. In a two-round Delphi survey, items with synonymous meanings were consolidated based on expert opinions (see Appendix A). Consequently, a 22-item pre-test version of the scale encompassing four dimensions was formed, and a five-point scoring system was determined by experts to collect participants’ responses. The first survey resulted in a 100% questionnaire return rate, a 0.900 authority coefficient, and a Kendall’s consistency coefficient of 0.672 (P＜0.05). The second survey yielded a corresponding 100% questionnaire recovery rate, a 0.900 authority coefficient, and a Kendall’s consistency coefficient of 0.724 (P＜0.05).

### The evaluation and exploration of the items

In the item analysis, the critical ratio of the items ranged from 5.233 to 18.096 (P < 0.05). The total scale’s Cronbach’s α coefficient was 0.891, but if items 11 (0.893) and 12 (0.892) were removed, the Cronbach’s α coefficient would increase. Additionally, the items-total correlation coefficients ranged from 0.423 to 0.710 (P < 0.05), except for items 11 (0.346) and 12 (0.389). In the exploratory factor analysis, all items showed factor loadings ranging from 0.622 to 0.887, except items 11 (0.290) and 12 (0.280). Given these results (Table 3), items 11 and 12 were removed from the draft, leading to the formation of a 20-item scale.

**Table 3:** Item analysis of the scale

Item	*t*-Value	*p*	Factor loading	Cronbach’s α if item deleted	Corrected item-total correlation coefficients	Retained item
1	11.932	＜0.001	0.812	0.886 (↓)	0.572^**^	√
2	6.887	＜0.001	0.666	0.890 (↓)	0.423^**^	√
3	14.241	＜0.001	0.622	0.885 (↓)	0.612^**^	√
4	12.844	＜0.001	0.851	0.885 (↓)	0.602^**^	√
5	10.003	＜0.001	0.751	0.889 (↓)	0.478^**^	√
6	12.804	＜0.001	0.839	0.885 (↓)	0.592^**^	√
7	9.965	＜0.001	0.844	0.888 (↓)	0.507^**^	√
8	9.300	＜0.001	0.872	0.888 (↓)	0.478^**^	√
9	14.278	＜0.001	0.887	0.884 (↓)	0.621^**^	√
10	7.751	＜0.001	0.824	0.890 (↓)	0.425^**^	√
11	5.233	＜0.001	0.290	0.893 (↑)	0.346^**^	×
12	14.555	＜0.001	0.857	0.883 (↓)	0.673^**^	√
13	10.504	＜0.001	0.848	0.887 (↓)	0.545^**^	√
14	10.611	＜0.001	0.810	0.887 (↓)	0.554^**^	√
15	15.817	＜0.001	0.819	0.883 (↓)	0.660^**^	√
16	10.478	＜0.001	0.872	0.886 (↓)	0.552^**^	√
17	15.130	＜0.001	0.826	0.884 (↓)	0.656^**^	√
18	14.504	＜0.001	0.837	0.884 (↓)	0.654^**^	√
19	15.433	＜0.001	0.836	0.883 (↓)	0.691^**^	√
20	18.096	＜0.001	0.817	0.882 (↓)	0.710^**^	√
21	10.659	＜0.001	0.655	0.889 (↓)	0.464^**^	√
22	5.732	＜0.001	0.280	0.892 (↑)	0.389^**^	×

Note. “√” indicates that the item was selected; ^**^ indicates significance p < 0.01; “↓” indicates that once the item is deleted, the

Cronbach’s α decreases

### The psychometric evaluation of the scale

#### Content validity

Seven experts who had not participated in the previous Delphi survey were invited to assess the content validity of the scale. As a result of the survey, the recovery rate of the consultation questionnaire was 1.000. The I-CVI ranged from 0.857 to 1.000, and the S-CVI was 0.986 (Appendix B).

#### Construct validity

**Fig. 2:**
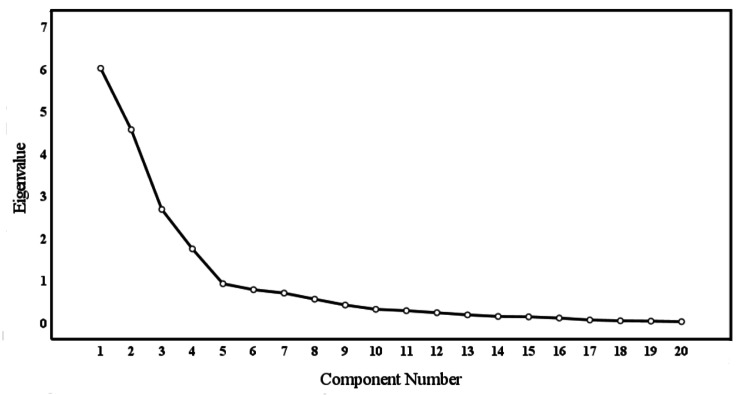
Screen plot of exploratory factor analysis for the home-based cardiac rehabilitation exercise adherence scale

In this study, the KMO value of 0.767 and significant Bartlett’s test of sphericity (χ^2^ = 2835.793, P < 0.001) indicated that the data was suitable for EFA. A maximum variance rotation was performed to extract common factors, resulting in four common factors with eigenvalues ≥ 1, which explained 75.1% of the total variation (Fig. 2). The factor loadings of the items were all greater than 0.4 and there were no cross-loadings (Table 4). The resulting four-factor structure, consisting of seeking supports, rehabilitation exercise, exercise monitoring, and information feedback, was consistent with previous theoretical expectations.

**Table 4:** Pattern matrix of the scale after the factor analysis

Item	Factor 1	Factor 2	Factor 3	Factor 4
1	0.039	***0.779***	0.145	0.080
2	0.041	***0.822***	0.077	0.080
3	0.103	***0.826***	0.289	0.047
4	0.022	***0.875***	0.232	0.040
5	0.141	***0.848***	0.194	0.017
6	***0.913***	0.108	0.077	0.157
7	***0.909***	0.107	0.192	0.109
8	***0.894***	0.084	0.124	0.131
9	***0.903***	0.125	0.141	0.065
10	***0.705***	0.144	0.027	0.123
11	0.194	0.045	0.118	***0.844***
12	0.110	0.099	0.064	***0.921***
13	0.261	0.130	0.114	***0.706***
14	0.026	0.132	0.066	***0.692***
15	0.083	0.154	0.013	***0.883***
16	0.077	0.230	***0.732***	0.017
17	0.168	0.150	***0.773***	0.068
18	0.057	0.168	***0.844***	0.152
19	0.214	0.208	***0.844***	0.215
20	0.059	0.158	***0.871***	0.110

Note. CFA: confirmatory factor analysis; EFA: exploratory factor analysis

**Fig. 3:**
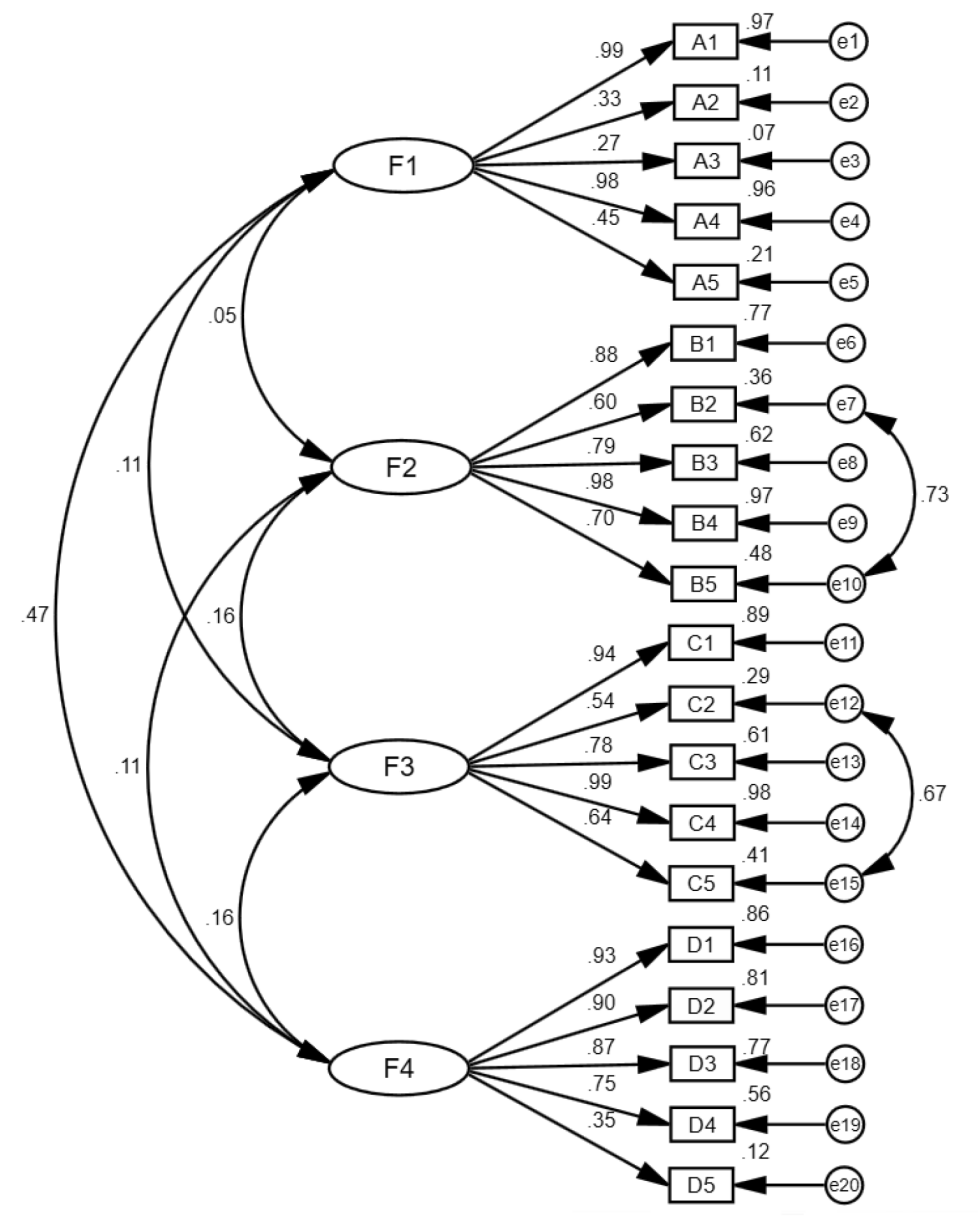
The four-factor model of the home-based cardiac rehabilitation exercise adherence scale. “F1” indicates seeking supports; “F2” indicates rehabilitation exercise; “F3” indicates exercise monitoring; “F4” indicates information feedback. A-D indicates specific items in different dimensions

In CFA, the initial structure model was modified twice based on the modification index, and the modified fitting indicators were examined (Fig. 3). The selected fitting indexes demonstrated that the four-factor structure scale had an appropriate fit, providing additional support for the consistency with the results of EFA and grounded theory. In the convergent validity analysis, the AVE values ranged from 0.48 to 0.64, and CR values ranged from 0.78 to 0.90. In assessing convergent validity, AVE values varied between 0.48 and 0.64, while CR values spanned from 0.78 to 0.90. In the discriminant validity analysis, the square root values of AVE fluctuated from 0.69 to 0.80, each exceeding the correlation coefficients of their respective factors (Table 5).

**Table 5:** Convergent validity and discriminant validity of the scale

**Factors**	Correlation between factors				**AVE**	**Sqrt (AVE)**	CR
	Factor 1	Factor 2	Factor 3	Factor 4			
Factor 1	1				0.48	0.69	0.78
Factor 2	0.045	1			0.64	0.80	0.90
Factor 3	0.105	0.159	1		0.64	0.80	0.89
Factor 4	0.471	0.108	0.157	1	0.63	0.79	0.89

Note. AVE: Average variance extracted; CR: Composite reliability

#### Calibration validity

As a calibration tool, the exercise self-efficacy scale was adopted to evaluate the calibration validity of the developed scale. The findings showed a highly positive correlation between the total score of the exercise self-efficacy scale and that of the developed scale (r = 0.726, P < 0.001).

#### Internal consistency reliability and test-retest reliability

The Cronbach’s α coefficient of the scale was 0.894, and the Cronbach’s α coefficients of each dimension ranged from 0.848 to 0.914. The split-half reliability coefficient of the scale was 0.695. After 2 weeks, 36 previously labeled patients with chronic heart failure were sampled to assess home-based cardiac rehabilitation exercise adherence, with a test-retest reliability coefficient of 0.745 (P < 0.001).

## Discussion

Among existing research tools, a scale for measuring home-based cardiac rehabilitation exercise adherence has not yet been explored. To address this gap, our study developed a grounded theory-driven evaluation tool, namely the home-based cardiac rehabilitation exercise adherence scale (Appendix C). We validated the four-factor structure of this scale, which overcomes the limitations of previous tools and comprehensively reflects adherence to home-based cardiac rehabilitation exercise. By incorporating this scale into a remote follow-up platform within medical institutions, we can actively and dynamically track patients’ home-based cardiac rehabilitation exercise adherence, significantly reducing the time cost of out-of-hospital follow-up for cardiac rehabilitation professionals. Additionally, this scale can multidimensional identify the weak links of patients’ home-based cardiac rehabilitation exercise adherence, facilitating cardiac rehabilitation professionals to develop precise intervention strategies.

Seeking supports is a crucial initial adherence behavior for patients with cardiovascular disease during home-based cardiac rehabilitation exercise [23]. This is particularly essential for patients with a low education level, who require educational supports from cardiac rehabilitation professionals [41]. Additionally, patients with cardiovascular disease significantly benefit from informational and familial supports in improving their home-based cardiac rehabilitation exercise skills and adherence [42, 43]. Thus, seeking support is a vital aspect in enhancing patients’ adherence to home-based cardiac rehabilitation exercise.

Exercise, as a recommended Level A1 evidence, is central to cardiac rehabilitation [4, 5]. It was confirmed that cardiac rehabilitation exercise has significant effects on cardiopulmonary function and clinical prognosis of patients with cardiovascular disease [44, 45]. Consequently, it is essential for patients to engage in home-based cardiac rehabilitation exercise. However, long-term adherence to cardiac rehabilitation exercise prescriptions remains a challenge for many patients with cardiovascular disease due to various obstacles [46]. The telehealth exercise-based cardiac rehabilitation models address this challenge to some extent with its intensity and variety of flexibility [47]. From a measurement perspective, the rehabilitation exercise dimension can directly and accurately evaluate patients’ home-based cardiac rehabilitation exercise adherence.

Exercise monitoring is a key adherence behavior for patients with cardiovascular disease during home-based cardiac rehabilitation exercises [23]. Effective exercise monitoring ensures the safety and effectiveness of patients’ cardiac rehabilitation routines [48]. There are two primary forms of exercise monitoring: tracking objective indicators and focusing on the subjective state. In home-based cardiac rehabilitation exercises, patients with high adherence to exercise monitoring can promptly identify exercise warnings and implement relevant interventions to prevent adverse cardiovascular events [49]. Therefore, exercise monitoring serves as an important index for assessing home-based cardiac rehabilitation exercise adherence.

Information feedback is a driving adherence behavior in cardiac rehabilitation exercises [23], which helps update exercise programs and guide exercise monitoring. Patients with cardiovascular disease provide monitoring information and subjective feelings to cardiac rehabilitation professionals, receiving strong supports and guidance during outpatient follow-ups [50]. Additionally, comprehensive information feedback enables cardiac rehabilitation professionals to make accurate clinical decisions and develop appropriate cardiac rehabilitation exercise programs for subsequent stages.

In general, based on previous grounded theory research [23], the dimensions of the scale were determined, with each dimension assigned to a different aspect for assessing home-based cardiac rehabilitation exercise adherence. The developed scale, covering four distinct aspects, can accurately and comprehensively evaluate the home-based cardiac rehabilitation exercise adherence of patients with cardiovascular disease, demonstrating good clinical practicability.

Based on grounded theory research and literature review, the dimensions and items of the scale were preliminarily determined. After a two-round Delphi survey, a pre-test version with 22 items was developed. The Delphi survey demonstrated satisfactory enthusiasm, authority, and consistency among the experts in relation to the items [25]. Thus, through the Delphi survey, the newly developed scale, driven by grounded theory, is deemed scientific and reasonable.

In the item analysis, the critical ratio of the items satisfies the reference standard value [27], supporting the appropriate discrimination of the scale. Except for items 11 and 22, the correlation coefficients of the item-total score were moderately to highly correlated, supporting the suitable applicability of the scale. Likewise, excluding items 11 and 22, the Cronbach’s α coefficient of the scale did not increase when items were deleted successively, implying the scale’s suitable homogeneity. Items 11 and 12 were subsequently excluded from the preliminary exploratory factor analysis. The remaining items, supported by factor loadings, demonstrated higher stability. Ultimately, based on the recommended standard values, items 11 and 22 were removed, leaving a total of 20 items in the item analysis.

In this study, the content validity, construct validity, and calibration validity of the scale were successively confirmed. Regarding content validity, both I-CVI and S-CVI exceeded the recommended standard values [29], supporting the scale’s appropriate content validity. In terms of construct validity, factor analysis was conducted. In the EFA, 20 items and four dimensions were extracted, explaining 75.1% of the total variation. In the CFA, a well-fitting model was obtained, with all fitting indexes in the acceptable range, signifying appropriate construct validity for the scale. Additionally, the acceptable AVE and CR values, along with the square root of AVE values being greater than the correlation coefficients, indicate that the scale possesses good convergent validity and discriminant validity [35]. As for calibration validity, the exercise self-efficacy scale was used as a calibration tool due to its previously demonstrated high correlation [51]. The scores of the exercise self-efficacy scale were significantly correlated with the scores of the newly developed scale, backing the scale’s suitable calibration validity. Overall, the home-based cardiac rehabilitation exercise adherence scale is both scientifically sound and demonstrates good validity.

In this study, the internal consistency reliability and test-retest reliability of the scale were confirmed. Regarding internal consistency reliability, both the Cronbach’s α coefficient and the split-half reliability coefficient of the scale exceeded the recommended reference values [37], supporting the scale’s proper internal consistency. Additionally, the test-retest reliability coefficient reached an appropriate range after the previously labeled participants were re-measured, affirming the scale’s measurement stability across time. In general, the home-based cardiac rehabilitation exercise adherence scale is scientifically sound and demonstrates good reliability.

### Limitations

There are some limitations to this study that warrant discussion. Firstly, bias resulting from the intrinsic nature of convenience sampling is unavoidable. Secondly, there were three items related to social support with factor loadings less than 0.40 in the CFA. However, we meticulously considered that social support is of vital importance in home-based cardiac rehabilitation exercise, and thus, all items were retained. Finally, the newly developed scale is applicable to all patients with cardiovascular diseases. However, the scale was only validated in patients with chronic heart failure in this study, which weakens the applicability and scientificity of the newly developed scale in other patients with cardiovascular diseases to a certain extent. Therefore, in future studies, the scale will be applied to other patients with cardiovascular disease to compensate for this limitation and improve the extrapolation of this scale.

## Conclusions

In the current study, we developed a home-based cardiac rehabilitation exercise adherence scale and validated its psychometric properties among patients with chronic heart failure. This newly developed scale covers four dimensions and 20 items. It can accurately and comprehensively evaluate the level of home-based cardiac rehabilitation exercise adherence for patients with cardiovascular disease. In future studies, the developed scale could be adopted to investigate adherence levels and measure the impact of interventions, thereby identifying weaknesses and evaluating their effectiveness.

### Electronic supplementary material

Below is the link to the electronic supplementary material.

Supplementary Material 1

Supplementary Material 2

Supplementary Material 3

## Data Availability

The datasets used and/or analysed during the current study are available from the corresponding author on reasonable request.

## Abbreviations

I-CVI: content validity index of the item
S-CVI: content validity index of the scale
EFA: exploratory factor analysis
KMO: Kaiser-Meyer-Olkin
CFA: confirmatory factor analysis
AVE: average variance extracted
CR: composite reliability
