# Supplementary material for: A scale for measuring home-based cardiac rehabilitation exercise adherence: a development and validation study
Source: BMC Nurs. 2023 Aug 7;22:259. doi: 10.1186/s12912-023-01426-2 (PMC10405489; doi:10.1186/s12912-023-01426-2)
Supplement: Supplementary file 1 — Supplementary Material 1 [file 12912_2023_1426_MOESM1_ESM.docx]

**Appendix A.** The results of items integration and optimization after a two-round Delphi survey

| **Dimensions** | **Initial items** | **Merged and optimized items** |
| --- | --- | --- |
| Seeking supports | I learned about cardiac rehabilitation exercise knowledge online. | **Integration and optimization:** I learned related knowledge and skills of cardiac rehabilitation exercise through various resources. |
|  | I got materials about cardiac rehabilitation exercises offline. |  |
|  | I attended lectures on cardiac rehabilitation exercise. |  |
|  | I participated in community cardiac rehabilitation exercise training. |  |
|  | I sought professional guidance on cardiac rehabilitation exercise monitoring skills. |  |
|  | I obtained self-management knowledge of cardiac rehabilitation exercise from professionals. |  |
|  | I contacted my peers to share my cardiac rehabilitation exercise experiences. | **Integration and optimization:** I contacted my peers to seek the experience and information supports of cardiac rehabilitation exercise. |
|  | I sought peer supports to complete cardiac rehabilitation exercise with more confidence. |  |
|  | I sought the help of my family to complete high-quality cardiac rehabilitation exercise. | **Integration and optimization:** I sought the supervision and supports of family to complete high-quality cardiac rehabilitation exercise. |
|  | I did cardiac rehabilitation exercise and monitoring under the supervision of my family. |  |
|  | I sought suggestions from professionals on preventing and controlling the adverse factors of cardiac rehabilitation exercise. | **The sentence is preserved.** |
|  | I sought professionals to regularly update my cardiac rehabilitation exercise prescription. | **The sentence is preserved.** |
| Rehabilitation exercise | I chose the appropriate cardiac rehabilitation exercise place according to the environment and preference. | **Integration and optimization:** I chose the appropriate cardiac rehabilitation exercise place based on environmental safety and personal preference. |
|  | I actively assessed whether the surrounding environment is suitable for cardiac rehabilitation exercise. |  |
|  | I actively evaluated whether self-condition is suitable for cardiac rehabilitation exercise. | **Integration and optimization:** I evaluated self-condition and physiological indicators before cardiac rehabilitation exercise. |
|  | I actively assessed basic physiological indicators using nearby devices. |  |
|  | I had medications ready for adverse cardiovascular events. | **The sentence was deleted.** |
|  | I did some related warm-up exercises before cardiac rehabilitation exercise. | **Integration and optimization:** I did related warm-up and relaxation exercises before and after cardiac rehabilitation exercise. |
|  | I did some related relaxation exercises after cardiac rehabilitation exercise. |  |
|  | I followed the cardiac rehabilitation exercise modes recommended by professionals. | **Integration and optimization:** I strictly followed the cardiac rehabilitation exercise program (mode, intensity, time, frequency) recommended by the professionals. |
|  | I followed cardiac rehabilitation exercise time recommended by professionals. |  |
|  | I followed cardiac rehabilitation exercise frequency recommended by my professionals. |  |
|  | I followed cardiac rehabilitation exercise intensity recommended by professionals. |  |
|  | I followed suggestions from professionals to increase my exercise load gradually. | **The sentence was preserved.** |
|  | I actively kept a regular journal of my cardiac rehabilitation exercise. | **The sentence was preserved.** |
| Exercise monitoring | I actively evaluated the subjective feelings during cardiac rehabilitation exercise. | **Integration and optimization:** I actively focused on subjective feelings and physiological indicators in cardiac rehabilitation exercise. |
|  | I made appropriate adjustments based on my subjective feelings during cardiac rehabilitation exercise. |  |
|  | I focused on the basic physiological indicators of cardiac rehabilitation exercise. |  |
|  | I judged the critical value of the relevant indicators during cardiac rehabilitation exercise. | **The sentence was deleted.** |
|  | I memorized the notes of cardiac rehabilitation and grasp the signs to stop exercising. | **The sentence was preserved.** |
|  | I skillfully used the monitoring tools of home cardiac rehabilitation exercise. | **The sentence was deleted.** |
|  | I was able to record and manage cardiac rehabilitation exercise data effectively. | **Revision:** I effectively recorded and managed the data generated by cardiac rehabilitation exercise. |
|  | I kept learning the knowledge and precautions of cardiac rehabilitation exercise monitoring. | **Integration and optimization:** The sentence was moved to the "Seeking Supports" and merged and optimized. |
|  | I followed the guidance from professionals for cardiac rehabilitation exercise monitoring. | **The sentence was preserved.** |
|  | I followed the suggestions of professionals for assessing exercise endurance regularly. | **The sentence was deleted.** |
|  | I contacted professionals when I felt uncomfortable during cardiac rehabilitation. | **The sentence was preserved.** |
| Information feedback | I regularly reported my subjective feelings about cardiac rehabilitation exercise to professionals. | **Integration and optimization:** I gave professionals feedback on the subjective feelings and weaknesses of cardiac rehabilitation exercise. |
|  | I reported to professionals on weaknesses in the management of cardiac rehabilitation exercises. |  |
|  | I expressed to professionals about the confusion I encountered in the cardiac rehabilitation exercises. |  |
|  | I regularly reported the monitoring information of cardiac rehabilitation exercise to the professionals. | **The sentence was preserved.** |
|  | I gave professionals feedback on cardiac function through clinical evaluation during follow-up visits. | **Integration and optimization:** I conducted regular outpatient follow-up and gave feedback on cardiac function by participating in clinical evaluation. |
|  | I followed the advice of professionals for regular outpatient reviews and follow-up visits. |  |
|  | I regularly conducted self-summary and feedback based on the data and feelings of cardiac rehabilitation exercise. | **The sentence was preserved.** |
|  | I told my family how I felt and what supportive behaviors I needed. | **Revision:** I gave feedback to my family about my feelings in cardiac rehabilitation exercise and the support behavior I needed. |
|  | I reported my views and experiences of cardiac rehabilitation to my peers. | **Revision:** I communicated with my peers and gave feedback on my views and experiences in cardiac rehabilitation exercise. |
