# Supplementary material for: A scale for measuring home-based cardiac rehabilitation exercise adherence: a development and validation study
Source: BMC Nurs. 2023 Aug 7;22:259. doi: 10.1186/s12912-023-01426-2 (PMC10405489; doi:10.1186/s12912-023-01426-2)
Supplement: Supplementary file 2 — Supplementary Material 2 [file 12912_2023_1426_MOESM2_ESM.docx]

**Appendix B.** Content validity analysis for the developed scale

| **Item** | **Experts (score)** | | | | | | | **I-CVI** | **S-CVI** |
| --- | --- | --- | --- | --- | --- | --- | --- | --- | --- |
|  | **1** | **2** | **3** | **4** | **5** | **6** | **7** |  |  |
| 1 | 4 | 3 | 4 | 3 | 4 | 4 | 3 | 1.000 | 0.986 |
| 2 | 2 | 4 | 4 | 4 | 3 | 4 | 3 | 0.857 |  |
| 3 | 4 | 4 | 4 | 4 | 4 | 3 | 4 | 1.000 |  |
| 4 | 4 | 4 | 3 | 3 | 4 | 3 | 3 | 1.000 |  |
| 5 | 3 | 4 | 4 | 4 | 4 | 4 | 3 | 1.000 |  |
| 6 | 4 | 3 | 4 | 4 | 4 | 3 | 3 | 1.000 |  |
| 7 | 4 | 4 | 3 | 3 | 3 | 4 | 4 | 1.000 |  |
| 8 | 4 | 4 | 4 | 3 | 4 | 3 | 3 | 1.000 |  |
| 9 | 3 | 4 | 4 | 4 | 3 | 4 | 4 | 1.000 |  |
| 10 | 4 | 4 | 4 | 4 | 3 | 4 | 4 | 1.000 |  |
| 11 | 4 | 4 | 3 | 4 | 4 | 3 | 3 | 1.000 |  |
| 12 | 4 | 3 | 4 | 3 | 4 | 4 | 4 | 1.000 |  |
| 13 | 4 | 4 | 4 | 4 | 3 | 3 | 4 | 1.000 |  |
| 14 | 4 | 4 | 4 | 2 | 4 | 3 | 3 | 0.857 |  |
| 15 | 3 | 4 | 4 | 4 | 3 | 4 | 4 | 1.000 |  |
| 16 | 4 | 3 | 4 | 3 | 4 | 3 | 3 | 1.000 |  |
| 17 | 4 | 4 | 3 | 4 | 4 | 4 | 3 | 1.000 |  |
| 18 | 3 | 4 | 4 | 4 | 4 | 3 | 3 | 1.000 |  |
| 19 | 4 | 4 | 4 | 3 | 4 | 4 | 4 | 1.000 |  |
| 20 | 3 | 4 | 4 | 4 | 3 | 4 | 3 | 1.000 |  |
