# Supplementary material for: A scale for measuring home-based cardiac rehabilitation exercise adherence: a development and validation study
Source: BMC Nurs. 2023 Aug 7;22:259. doi: 10.1186/s12912-023-01426-2 (PMC10405489; doi:10.1186/s12912-023-01426-2)
Supplement: Supplementary file 3 — Supplementary Material 3 [file 12912_2023_1426_MOESM3_ESM.docx]

**Appendix C.** Home-based cardiac rehabilitation exercise adherence scale

| **Dimensions** | **Items** | **Strongly agree** | **Agree** | **Neutral** | **Disagree** | **Strongly disagree** |
| --- | --- | --- | --- | --- | --- | --- |
| Seeking supports | I learned related knowledge and skills of cardiac rehabilitation exercise through various resources. | 5 | 4 | 3 | 2 | 1 |
|  | I contacted my peers to seek the experience and information supports of cardiac rehabilitation exercise. | 5 | 4 | 3 | 2 | 1 |
|  | I sought the supervision and supports of family to complete high-quality cardiac rehabilitation exercise. | 5 | 4 | 3 | 2 | 1 |
|  | I sought suggestions from professionals on preventing and controlling the adverse factors of cardiac rehabilitation exercise. | 5 | 4 | 3 | 2 | 1 |
|  | I sought professionals to regularly update my cardiac rehabilitation exercise prescription. | 5 | 4 | 3 | 2 | 1 |
| Rehabilitation exercise | I chose the appropriate cardiac rehabilitation exercise place based on environmental safety and personal preference. | 5 | 4 | 3 | 2 | 1 |
|  | I evaluated self-condition and physiological indicators before cardiac rehabilitation exercise. | 5 | 4 | 3 | 2 | 1 |
|  | I did related warm-up and relaxation exercises before and after cardiac rehabilitation exercise. | 5 | 4 | 3 | 2 | 1 |
|  | I strictly followed the cardiac rehabilitation exercise program (mode, intensity, time, frequency) recommended by the professionals. | 5 | 4 | 3 | 2 | 1 |
|  | I followed suggestions from professionals to increase my exercise load gradually. | 5 | 4 | 3 | 2 | 1 |
|  | I actively kept a regular journal of my cardiac rehabilitation exercise. | 5 | 4 | 3 | 2 | 1 |
| Exercise monitoring | I actively focused on subjective feelings and physiological indicators in cardiac rehabilitation exercise. | 5 | 4 | 3 | 2 | 1 |
|  | I memorized the notes of cardiac rehabilitation and grasp the signs to stop exercising. | 5 | 4 | 3 | 2 | 1 |
|  | I effectively recorded and managed the data generated by cardiac rehabilitation exercise. | 5 | 4 | 3 | 2 | 1 |
|  | I followed the guidance from professionals for cardiac rehabilitation exercise monitoring. | 5 | 4 | 3 | 2 | 1 |
|  | I contacted professionals when I felt uncomfortable during cardiac rehabilitation. | 5 | 4 | 3 | 2 | 1 |
| Information feedback | I gave professionals feedback on the subjective feelings and weaknesses of cardiac rehabilitation exercise. | 5 | 4 | 3 | 2 | 1 |
|  | I regularly reported the monitoring information of cardiac rehabilitation exercise to the professionals. | 5 | 4 | 3 | 2 | 1 |
|  | I conducted regular outpatient follow-up and gave feedback on cardiac function by participating in clinical evaluation. | 5 | 4 | 3 | 2 | 1 |
|  | I regularly conducted self-summary and feedback based on the data and feelings of cardiac rehabilitation exercise. | 5 | 4 | 3 | 2 | 1 |
|  | I gave feedback to my family about my feelings in cardiac rehabilitation exercise and the support behavior I needed. | 5 | 4 | 3 | 2 | 1 |
|  | I communicated with my peers and gave feedback on my views and experiences in cardiac rehabilitation exercise. | 5 | 4 | 3 | 2 | 1 |
